# Dr. R. A. T. Ridley

**Published:** 1872-02

**Authors:** 


Dr. R. A. T. Ridley.—We are deeply pained to note the death of our special friend, Dr. R. A. T. Ridley, of LaGrange. He was a gentleman of marked ability in his profession, and distinguished as an eminent citizen of Georgia.

